# Fine-Scale Population Structure but Limited Genetic Differentiation in a Cooperatively Breeding Paper Wasp

**DOI:** 10.1093/gbe/evaa070

**Published:** 2020-04-09

**Authors:** Sarah E Bluher, Sara E Miller, Michael J Sheehan

**Affiliations:** Department of Neurobiology and Behavior, Cornell University

**Keywords:** social insects, Hymenoptera, isolation by distance, cooperation, dispersal

## Abstract

Relatively little is known about the processes shaping population structure in cooperatively breeding insect species, despite the long-hypothesized importance of population structure in shaping patterns of cooperative breeding. *Polistes* paper wasps are primitively eusocial insects, with a cooperative breeding system in which females often found nests in cooperative associations. Prior mark-recapture studies of *Polistes* have documented extreme female philopatry, although genetic studies frequently fail to detect the strong population structure expected for highly philopatric species. Together these findings have led to lack of consensus on the degree of dispersal and population structure in these species. This study assessed population structure of female *Polistes fuscatus* wasps at three scales: within a single site, throughout Central New York, and across the Northeastern United States. Patterns of spatial genetic clustering and isolation by distance were observed in nuclear and mitochondrial genomes at the continental scale. Remarkably, population structure was evident even at fine spatial scales within a single collection site. However, *P. fuscatus* had low levels of genetic differentiation across long distances. These results suggest that *P. fuscatus* wasps may employ multiple dispersal strategies, including extreme natal philopatry as well as longer-distance dispersal. We observed greater genetic differentiation in mitochondrial genes than in the nuclear genome, indicative of increased dispersal distances in males. Our findings support the hypothesis that limited female dispersal contributes toward population structure in paper wasps.

## Introduction

The genetic structure of populations is shaped by selection, genetic drift, and gene flow (Hartl and Clark 1997). Localized directional selection and random genetic drift cause divergence between subpopulations, whereas stabilizing selection and migration between subpopulations induce homogeneity (Hartl and Clark 1997). Social and mating systems may shape patterns of dispersal and influence population structure (Hatchwell 2009). Concurrently, patterns of dispersal and population genetic structure may also influence social and mating systems by determining the spatial availability of kin or suitable mates (Greenwood 1980). Given the importance of population structure to social evolution theory (e.g., Hamilton 1964; Hochberg et al. 2008; Platt and Bever 2009), it is surprising that relatively little is known about the structure of populations of cooperatively breeding social Hymenoptera.

Cooperative breeding occurs when individuals work together to raise offspring, with nonbreeding individuals often sacrificing direct fitness for indirect fitness (Emlen and Oring 1977; Jennions and Macdonald 1994; Reeve et al. 2000; Clutton-Brock 2002). Increasing the relatedness between neighboring individuals has been predicted to facilitate the evolution of cooperative behavior (Hamilton 1964). Theories of social evolution predict limited dispersal in cooperatively breeding species; the feedback between cooperation and dispersal is expected to strengthen structure in subpopulations, leading to a pattern of isolation by distance (IBD) (Platt and Bever 2009). Studies testing these predictions within cooperatively breeding taxa have reported philopatry (Radespiel et al. 2003; Handley and Perrin 2007; Sharp et al. 2008), fine-scale genetic structure (Storz 1999; Painter et al. 2000; Radespiel et al. 2001; Covas et al. 2006), and IBD (Sundström et al. 2003; Clémencet et al. 2005). However, similar evidence for philopatry and genetic structure has also been found in noncooperative taxa (Paradis et al. 1998; Shorey et al. 2000; Piertney et al. 2008; Bretman et al. 2011), suggesting that dispersal limitation can result from mechanisms unrelated to cooperation. Additionally, recent studies have reported both low and high degrees of population structure among social insect subpopulations (e.g., Schlüns et al. 2009; Goulson et al. 2011; Jowers et al. 2013; Duarte et al. 2014; Johansson et al. 2018), raising questions regarding the ubiquity of reduced dispersal in cooperatively breeding species. Thus, a major empirical challenge for studies of social evolution is to document patterns of population genetic structure in cooperatively breeding taxa to help understand the relationship between social systems and population structure.

Paper wasps in the genus *Polistes* are primitively eusocial insects and are an emerging model system for testing theoretical predictions of social evolution (Jandt et al. 2014), making this an attractive system to assess population structure. In the temperate zone, *Polistes* reproductive females, known as gynes, emerge and mate in the fall, before overwintering in hibernacula prior to the spring nesting season. *Polistes* displays a wide range of cooperative behavior within and among species (Sheehan et al. 2015; Miller, Bluher, et al. 2018). New nests are initiated each spring by either a single female foundress or a small group of cooperating foundresses (Eberhard 1969; Metcalf and Whitt 1977; Gamboa et al. 1978; Gibo 1978; Klahn 1988). Individuals that fail to found independent nests often join foundress associations (Lorenzi and Cervo 1995; Cervo and Lorenzi 1996; Zanette and Field 2011; Wright et al. 2019). Within cooperative associations, reproduction follows a linear dominance hierarchy in which the dominant foundress lays the majority of eggs and often consumes those laid by her subordinates (West 1967; Strassmann 1981; Röseler et al. 1986; Pratte 1989; Jandt et al. 2014). Subordinate foundresses help to provision the offspring of the dominant female and contribute disproportionately to risky foraging efforts (Gamboa et al. 1978; de Souza et al 2008). *Polistes* foundresses therefore must decide how far to disperse during predictably timed dispersal events in the fall and in the spring. Cooperative foundresses can increase inclusive fitness by founding or joining nests with relatives. Given that relatives are more likely to co-occur proximal to their natal nests, this leads to the prediction that cooperatively breeding wasps will be highly philopatric, resulting in elevated inbreeding and a genetic signal of IBD.

Supporting the prediction of high rates of philopatry, mark-recapture studies have shown that female *Polistes* wasps often stay very close to their natal territories. For example, when females marked on their nests in the fall are located again the next spring, they tend to be found within a few dozen meters of their natal nest (Klahn 1979; Cervo and Turillazzi 1985; Gamboa et al. 1986; Sheehan et al. 2017). Proximity to the natal nest has been proposed to be a major factor promoting cooperation among related females in cofoundress associations. Indeed, larger cofoundress associations tend to be found closer to former nest sites compared with their less cooperative counterparts (Klahn 1979). A study of *P. chinensis* populations in Japan and New Zealand predicted mean axial parent–offspring dispersal distances of 33–60 m (Tsuchida et al. 2014). The dearth of recorded dispersal events beyond a few dozen meters has encouraged the notion that female paper wasps are highly philopatric. However, given the difficulty of rediscovering highly dispersed individuals, mark-recapture studies may be biased toward shorter dispersal distances. Additionally, very little is known about male dispersal in paper wasps, although patterns of male-biased gene flow have been observed in several species of eusocial Hymenoptera (Ross and Shoemaker 1997; Clarke et al. 2002; Doums et al. 2002; Rüppell et al. 2003; Sundström et al. 2003). Studies of population genetic structure can therefore provide a more robust window into overall dispersal patterns.

Measurements of genetic differentiation and inbreeding have widely varied among populations of *Polistes* studied to date (supplementary table S1, Supplementary Material online). Populations of *Polistes exclamans*, *P. metricus*, and *P. bellicosus* have inbreeding coefficients significantly higher than 0 (Davis et al. 1990), but studies of *P. carolina*, *P. jadwigae*, *P. chinensis*, *P. biglumis*, *P. canadensis*, and *P. dominula* did not detect any significant genetic signature of inbreeding (Davis et al. 1990; Tsuchida 1994; Miyano and Hasegawa 1998; Johnson and Starks 2004; Seppä et al. 2011; Lengronne et al. 2012). Of the studies that have investigated IBD in *Polistes*, half detected significant patterns of IBD (*P. dominula*, *P. chinensis*, and *P. nimpha*), whereas the remaining studies detected no significant IBD (*P. dominula*, *P. canadensis*, *P. olivaceus*, *Polistes fuscatus*, *P. metricus*, and *P. dorsalis*) (Johnson and Starks 2004; Lengronne et al. 2012; Uddin and Tsuchida 2012; Tsuchida et al. 2014; Kozyra et al. 2015; Miller et al. 2020). The two studies of IBD in *P. dominula* varied dramatically in scale with significant IBD detected in the small-scale study (*r*_P_ = 0.2 km) (Lengronne et al. 2012), but not in the large-scale study (*r*_p_ = 240 km) (Johnson and Starks 2004). Taken together, these studies have resulted in a lack of consensus on population structure in paper wasps.

There are two important caveats when interpreting past studies of genetic structure in *Polistes*. First, authors have defined subpopulations using a variety of spatial scales, with a lack of cross-scale analyses. Dispersal rates are typically measured at a single spatial scale within a given study, and these scales vary widely across studies (e.g., Davis et al. 1990; Tsuchida et al. 2014), making comparisons between studies less robust. Assuming equal degrees of population structure, *F*_IS_ values are expected to scale with subpopulation size due to the fine-scale structure nested within more broadly defined subpopulations, whereas *F*_ST_ values and IBD depend on both the scale of and the distance between subpopulations. Considering genetic structure across multiple scales is important, as smaller distances potentially provide information on recent local dispersal events, whereas larger geographic distances tend to incorporate broader dispersal trends across habitats and generations. Second, with the exception of Miller et al. (2020), which used whole-genome resequence data to document low *F*_ST_ between *P. fuscatus* populations in New York and Massachusetts, all studies have inferred population structure from a limited number of genetic markers using microsatellites or allozymes. Failures to detect population structure in these studies may be caused by insufficient genetic variation to detect structure at fine spatial scales. Comparative analyses of the power of allozymes, microsatellites, and single-nucleotide polymorphisms (SNPs) to detect population structure have found that the three type of loci yield similar estimates of genetic divergence (Smith et al. 2007; Fischer et al. 2017; Muñoz et al. 2017; Lemopoulos et al. 2019). However, SNPs provide a more accurate estimate of heterozygosity across the genome (Lamaze et al. 2012; Fischer et al 2017) and are more sensitive for identifying family relatedness and introgression (Lamaze et al. 2012; Muñoz et al. 2017; Lemopoulos et al. 2019). Therefore, clarifying population structure in *Polistes* wasps will require comparisons of large numbers of genetic markers across multiple spatial scales.

In this study, we investigated population structure in the paper wasp *P. fuscatus*. Whole-genome resequencing data were generated for 204 female *P. fuscatus* collected in the Eastern United States, making this study among the most comprehensive tests of genetic population structure in a social insect species to date. We tested for genetic differentiation and IBD among populations at three spatial scales: within a single site, within Central New York, and across the Eastern United States.

## Materials and Methods

### Sampling

Female *P. fuscatus* individuals were collected from nests and on the wing throughout the Ithaca region in central New York (*N* = 182) in 2015 and 2016 (supplementary table S2, Supplementary Material online). For nests collected from cabins in Arnot Forest (Van Etten, New York), we recorded detailed information regarding nest placement. Female *P. fuscatus* were additionally collected in 2015–2017 from northern New York (*N* = 5), Massachusetts (*N* = 11), Maryland (*N* = 8) and North Carolina (*N* = 7) to measure genetic differentiation across longer distances. We approximated the size of each region by using the Google Earth measurement tool (earth.google.com) to trace a polygon around sampling sites and estimate the radii of this polygon. Only one individual per nest was sequenced in order to avoid confounding the effects of nestmate relatedness with patterns of broad population structure. To account for related individuals caught on the wing, we removed one individual from each pair of samples with kinship coefficients >0.1 (see below). We removed eight individuals from Central New York and one individual from North Carolina resulting in a final data set of *N* = 174 individuals and *N* = 6 individuals for these populations, respectively.

### Whole-Genome Resequencing and Variant Detection

DNA was extracted from a single leg for each individual using the Qiagen Puregene Core Kit A. Paired-end whole-genome libraries were prepared with an average insert size of 550 bp using the Nextera library preparation kit, first shearing DNA with the Covaris S2 Adaptive Focused Acoustic Disruptor (Covaris, Inc.). Library sizes were quantified using a bioanalyzer (Agilent Genomics, Santa Clara, CA) prior to being sequenced on the Illumina HiSeq 2000. Raw reads were processed with Trimmomatic (v0.36) to remove adaptors and poor-quality sequence. Trimmed reads were mapped to the *P. fuscatus* reference genome, which included the mtDNA reference genome (Miller et al. 2020) using the Burrows–Wheeler Aligner (v0.7.13) (Li and Durbin 2010). Variants were identified with Picard tools (v2.8.2) (http://broadinstitute.github.io/picard; last accessed April 2020) in combination with the HaplotypeCaller tool in GATK (v3.8) (Van der Auwera et al. 2013). Variant calls for each individual were merged in GATK using the GenotypeGVCF tool, which aggregates variant information across samples to correct genotype likelihoods and improve the confidence of SNP identification. After alignment, SNPs were hard filtered with GATK to remove poor-quality variants using the parameters: strand bias >60, strand odds ratio >3.0, and RMS mapping quality <40.0. Resequenced genomes had an average coverage of 6× (supplementary table S2, Supplementary Material online). Related individuals were identified using the –relatedness2 option in VCFtools (v0.1.15) (Danecek et al. 2011) and removed from the data set. The initial data set of 9.58 million SNPs was further filtered with VCFtools to exclude SNPs with poor coverage across individuals using the option –max-missing 0.8, resulting in a reduced data set of 4.71 million SNPs. Lastly, singleton SNPs can confound model-based estimates of population structure (Linck and Battey 2019). To account for regions missing data among individuals, we filtered by minor allele count using the option –mac 3 in VCFtools to avoid removing different site frequency spectrum classes across genomic regions with different sequencing coverage (Linck and Battey 2019). This allele count cutoff is similar to a minor allele frequency cutoff of <0.01. The final filtered data set contains 1.56 million SNPs.

### Descriptive Statistics of Populations

Mean whole-genome nucleotide diversity (*π*), inbreeding coefficients (*F*_IS_), expected heterozygosity (*H*_e_) and observed heterozygosity (*H*_o_) were calculated for the entire Eastern US population and each of the regionally defined subpopulations in 10,000-bp windows with VCFtools (Danecek et al. 2011) and vcflib (v1.0.0-rc2) (https://github.com/ekg/vcflib; last accessed April 2020).

We calculated mitochondrial nucleotide diversity (*π*_mt_) by concatenating the mitochondrial genes COI, COII, ATP6, ATP8, ND4, ND5, 12S, 16S, and CytB and identifying variants within the concatenated sequence. Concatenated mitochondrial genes have been shown to be as effective as whole mitochondrial genome sequences for detecting population structure using a similar set of mitochondrial genes in *Apis mellifera* (Eimanifar et al. 2018).

Due to differences in sample size between locations, we tested for the effect of sample size on estimates of population genetics statistics by randomly subsampling eight individuals from Central New York and Massachusetts to yield a more even distribution of samples across regions and sites for comparisons across the Eastern United States.

### General Patterns of Population Structure

We visualized population separation at three different scales: across the Eastern United States, within Central New York, and at a single collection site within Central New York, Arnot Forest (Van Etten, New York). We used multidimensional scaling (MDS) to plot genetic relationships based on estimates of pairwise genetic differentiation among all 204 individuals from the Eastern United States. MDS plots were generated using plink (v1.07), specifying only the top ten axes (–mds-plot 10). Additionally, for comparisons across the Eastern United States, to avoid overrepresentation of individuals from highly sampled areas, we used the same data set of subsampled individuals from Central New York and Massachusetts described above. For comparisons within Central New York, we similarly randomly subsampled individuals from Freeville and Arnot Forest.

Genetic structure across the Eastern United States was examined by using fastSTRUCTURE (v1.0) to determine the optimal number of demes within the data (Raj et al. 2014). To avoid genetic linkage among markers, the filtered data set was thinned to include only one biallelic SNP every 10,000 bp. This resulted in a data set of 20,633 SNPs after filtering. We ran fastSTRUCTURE for *K* = 1–6 demes and determined the optimal *K* value with the “chooseK” script included with the program. We iterated the program 50 times and merged the runs with CLUMPP (v1.1.2) (Jakobsson and Rosenberg 2007).

To explore the repeatability of our findings, we additionally assessed population structure using the program STRUCTURE (v2.3.4) (Pritchard et al. 2000). Due to the computational demands of the program, we filtered our data to include one biallelic SNP every 50,000 bp resulting in a smaller data set of 4,385 SNPs. STRUCTURE can underestimate the true number of subpopulations when samples are unevenly distributed (Puechmaille 2016), therefore we used the same downsampled data set of individuals from the Eastern United States from the MDS analysis. We ran 50 replicates for *K* = 1–6 using a burnin period of 100,000 steps followed by 200,000 sampling steps implemented with Structure_threader (v1.2.11) (Pina-Martins et al. 2017). Repeated runs were processed with Structure Harvester (Earl and vonHoldt 2012) and merged with CLUMPP (Jakobsson and Rosenberg 2007).

### Isolation by Distance

To quantify the extent of IBD, we followed Rousset’s method of inferring average dispersal rates using the slope of the linear matrix regression of pairwise *F*_ST_/(1 – *F*_ST_) on geographical distance (Rousset 1997). Calculations of *F*_ST_ were made using all samples from each region. This one-dimensional regression is appropriate for analyzing data along transects and yields the following predicted relationship between the mean squared parent–offspring axial dispersal distance ($\sigma^{2}$) and the regression slope (*b*):

$$\sigma^{2}=\frac{1}{4\pi D_{e}b}$$

where *D*_e_ is the effective population density of breeders. The only formal assessment of *Polistes* nest density (a good approximation of *D*_e_) is 2,500 nests/km^2^, calculated for *P. chinensis* from Japan (Tsuchida et al. 2014). To estimate *D*_e_ in *P. fuscatus*, we intensively surveyed the number of *P. fuscatus* nests in Arnot Forest, yielding an estimated nest density of 1,080 nests/km^2^. However, less formal surveys of *P. fuscatus* nest density throughout Central New York suggest that Arnot Forest may have an unusually high nest density relative to nearby regions. We calculated $\sigma^{2}$ using estimates of nest density from *P. fuscatus* in Arnot Forest but note that an overestimate of *D*_e_ will likely underestimate dispersal distance. Additionally, Rousset’s method was developed for diploid populations. On average, haplodiploid alleles should coalesce at three-fourths of the rate of diploid alleles. Faster coalescence will affect *b*, likely leading to an overestimate of dispersal distance. Dispersal reconstructions calculated using the above equation assume constant mutation rate, migration rate, and effective population density over time.

### Sex-Biased Dispersal

Studies of mitochondrial and nuclear population structure have revealed male-biased gene flow patterns in several species of eusocial Hymenoptera (Ross and Shoemaker 1997; Clarke et al. 2002; Doums et al. 2002; Rüppell et al. 2003; Sundström et al. 2003). Male-biased dispersal and female philopatry can result in higher genetic differentiation between subpopulations for maternally inherited mitochondrial genes than for the nuclear genome (Prugnolle and de Meeus 2002). Sex-biased dispersal can be assessed by comparing nuclear (*F*_ST(nuc)_) and mitochondrial (*F*_ST(mito)_) genetic diversity statistics. Under equal male and female dispersal, *F*_ST(mito)_ and *F*_ST(nuc)_ have the following relationship (Crochet 2000):

$$F_{\mathrm{ST}(\mathrm{mito})}=\;\frac{4F_{\mathrm{ST}(\mathrm{nuc})}}{1+3F_{\mathrm{ST}(\mathrm{nuc})}}.$$

Male-based dispersal is supported when observed *F*_ST(mito)_ values are greater than expected values.

Pairwise *F*_ST(mito)_ and *F*_ST(nuc)_ between subpopulations were calculated using VCFtools. The strength and significance of observed patterns of IBD were analyzed using linear models.

### Mitochondrial Haplotype Network

To visualize mitochondrial population structure at multiple scales, haplotype networks were constructed using the haploNet function from the R package pegas (Paradis 2010). Mitochondrial variants were identified from the concatenated mitochondrial genes described above. For the mitochondrial haplotype network of the Eastern United States and Central New York, we used the same subsampled data sets described above. For the mitochondrial haplotype network of individuals collected within Arnot Forest, we restricted our sampling to individuals collected from nests on cabins (excluding those caught flying on the wing). One individual (Fuscatus_NY_16-27) was excluded due to low coverage of mitochondrial sequence. This resulted in a final data set of 50 individuals.

## Results

### Population Genetics Statistics

Across the Eastern United States, all populations had inbreeding coefficients (*F*_IS_) significantly >0. The average *F*_IS_ was 0.296 (table 1), consistent with a high degree of inbreeding in these populations. However, positive *F*_IS_ values could also result from subpopulation structure within populations, known as the Wahlund effect (Wahlund 1928; Sinnock 1975). Mean whole-genome nucleotide diversity (*π*) was similar across populations (range: 0.0005–0.0008). Central New York and Massachusetts showed a slight decrease in mean nucleotide diversity of mitochondrial genes (*π*_mt_) relative to the other populations. All population genetic statistics were similar between the full and the downsampled data sets.

**Table 1:** Population Genetic Statistics for Each Study Region and across All Regions in Eastern United States

Location	Latitude	Longitude	*r* (km)	*N*	*π*	*π*_mt_	*H*_e_	*H*_o_	*F*_IS_
C. NY (all)	42	−77	20	174	0.0007	0.0025	0.0961	0.0718	0.2748
C. NY (subset)	42	−77	20	8	0.0007	0.0038	0.0935	0.0822	0.2500
N. NY	43	−74	2.2	5	0.0008	0.0070	0.0814	0.0837	0.0802
NC	36	−79	3.5	6	0.0007	0.0077	0.0808	0.0681	0.2296
MD	39	−77	67	8	0.0005	0.0072	0.0742	0.0485	0.4249
MA (all)	42	−71	15	11	0.0006	0.0027	0.0799	0.0618	0.2745
MA (subset)	42	−71	15	8	0.0007	0.0038	0.0806	0.0667	0.2223
All regions (all)	NA	NA	750	204	0.0007	0.0026	0.0961	0.0706	0.2858
All regions (subset)	NA	NA	750	33	0.0007	0.0039	0.0936	0.0691	0.2955

Note.—*r*, radius of specified population in kilometers; *N*, number of individuals; *π*_mt_, mitochondrial gene nucleotide diversity; *π*, mean whole-genome nucleotide diversity; *F*_IS_, inbreeding coefficient.

### Population Structure

**Fig. 1.**
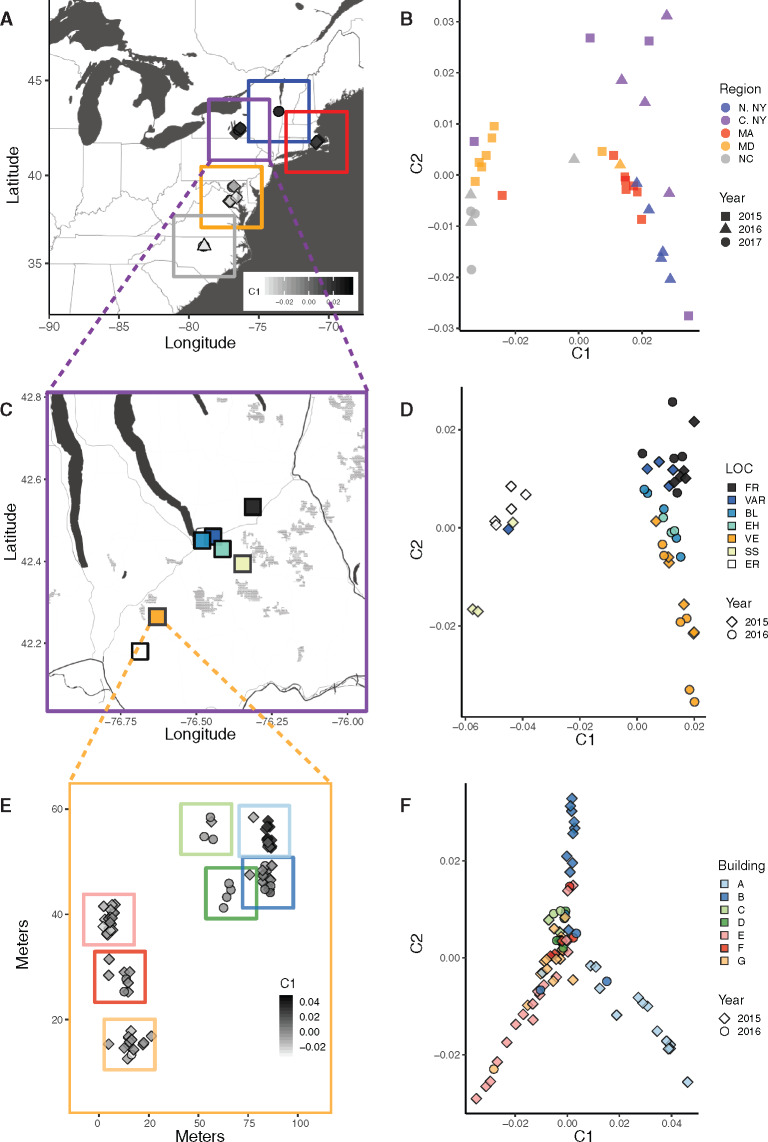
—Plots of the first two axes from MDS analyses of whole-genome sequences reveal population genetic structure at three different spatial scales. (*A*) Location of all sampling sites across the Eastern United States. (*B*) MDS plot of individuals collected across the Eastern United States. Populations have been subsampled to include the same number of individuals across regions. (*C*) Inset showing sampling sites within the Central New York region. (*D*) MDS plot of Central New York individuals. Sample locations are indicated by color. (*E*) Inset showing sampling sites within Arnot Forest in Van Etten New York with approximate location of building indicated by the colored boxes. (*F*) MDS plot of individuals within Arnot Forest. The color of points corresponds to the building where the individual was collected. Sampling locations and abbreviations are given in supplementary table S2, Supplementary Material online. Eigenvalues are given in supplementary table S3, Supplementary Material online.

MDS plots grouped *P. fuscatus* individuals by region at the three different spatial scales. Across the Eastern United States, the first two MDS axes (C1 and C2) grouped individuals broadly by region, with some overlap among regions (fig. 1*A* and *B*). Within central New York (fig. 1*C* and *D*), C1 separates individuals collected from Erin and Slaterville Springs from the other populations, whereas C2 roughly correlates with latitude. Remarkably, for *P. fuscatus* within the same site in Arnot Forest, the C1 axis largely separated individuals nesting on buildings A–B from individuals nesting on buildings E–G, with individuals nesting on buildings C–D showing intermediate values (fig. 1*E* and *F*). Individuals sampled across multiple years clustered by location across the Eastern United States and within Central New York. Within Arnot Forest, individuals sampled in 2016 tended to favor the same general location as those sampled in 2015. In several cases, closely related individuals nested on the same building across multiple years, suggesting the possibility of fine-scale philopatry for some foundresses. Eigenvalues for all MDS plots are given in supplementary table S3, Supplementary Material online.

**Fig. 2.**
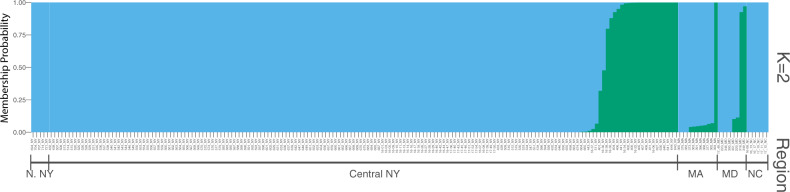
—Cluster analysis of populations using fastSTRUCTURE for the most highly supported model *K* = 2. Color within a column indicates the inferred posterior probability that the individual is a member of a particular cluster. Each individual is represented by a single column. Regions represented are Northern New York (N. NY), central New York, Massachusetts (MA), Maryland (MD), and North Carolina (NC).

In contrast to the fine-scale spatial segregation detected in MDS plots, population differentiation among regions was not observed in the fastSTRUCTURE analysis (fig. 2). The best-supported model was *K* = 1 or *K* = 2. The model output for *K* = 2 demes indicates near panmictic levels of homogeneity for most individuals, but with a few individuals from Central New York, Massachusetts, and Maryland assigned to a second population. Model outputs for *K* = 3–6 showed no evidence of regional population differentiation although the prediction of a second and third population within the data remains consistent with increasing values of *K* (supplementary fig. S1, Supplementary Material online). Similar results were obtained analyzing data with STRUCTURE (supplementary fig. S2, Supplementary Material online).

### Isolation by Distance

**Fig. 3.**
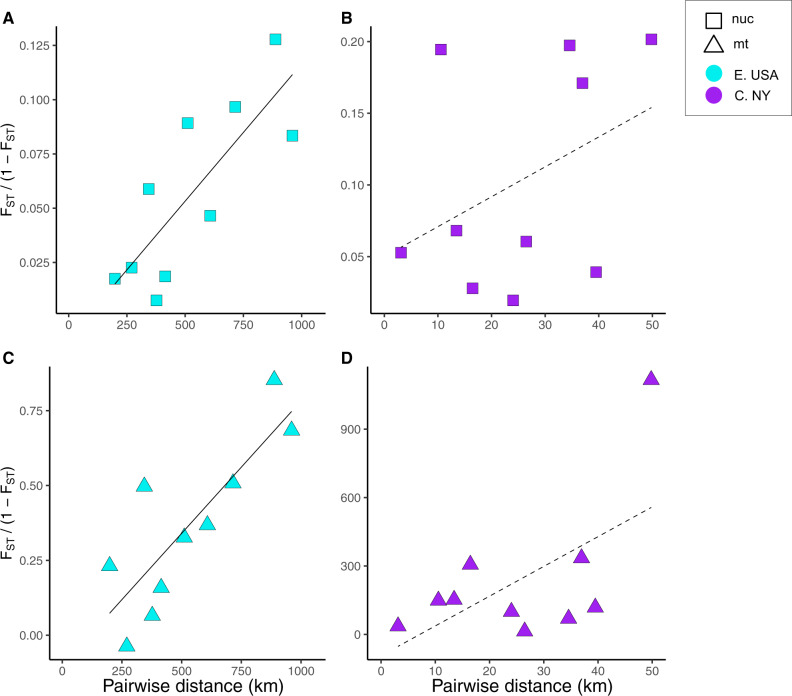
—Linearized pairwise whole-genome divergence versus geographic distance across the Eastern United States (*A*) and within the Central New York region (C. NY) (*B*) and pairwise mitochondrial divergence versus geographic distance across the Eastern United States (*C*) and within the Central New York region (*D*). Patterns of IBD are evident at both scales and for both types of markers. Nonsignificant regressions are illustrated with dotted lines.

Whole-genome analyses revealed significant patterns of IBD in *P. fuscatus* wasps both across the Eastern United States and within Central New York (fig. 3*A* and *B*). Linear models showed significant correlation between linearized genetic and geographic distance across the Eastern United States (*y* = 1.27 × 10^−4^*x* – 0.01, *R*^2^ = 0.60, *P* < 0.005) but a poor correlation between these values in Central New York (*y* = 0.0021*x* – 0.05, *R*^2^ = 0.05, *P* = 0.26). The mean pairwise divergence across all subpopulation comparisons was *F*_ST_ = 0.0525 (pairwise comparisons given in supplementary table S4, Supplementary Material online). Results from mitochondrial data were similar (fig. 3*C* and *D*) with significant patterns of IBD detected in mitochondrial sequence across the Eastern United States (*y* = 8.8 × 10^−4^*x* – 0.1, *R*^2^ =0.64, *P* = 0.003) and a weaker correlation within Central New York (*y* = 0.13*x* − 0.93, *R*^2^ = 0.27, *P* = 0.072). The mean pairwise mitochondrial divergence across all comparisons was *F*_ST_ = 0.24 (pairwise comparisons given in supplementary table S4, Supplementary Material online).

**Fig. 4.**
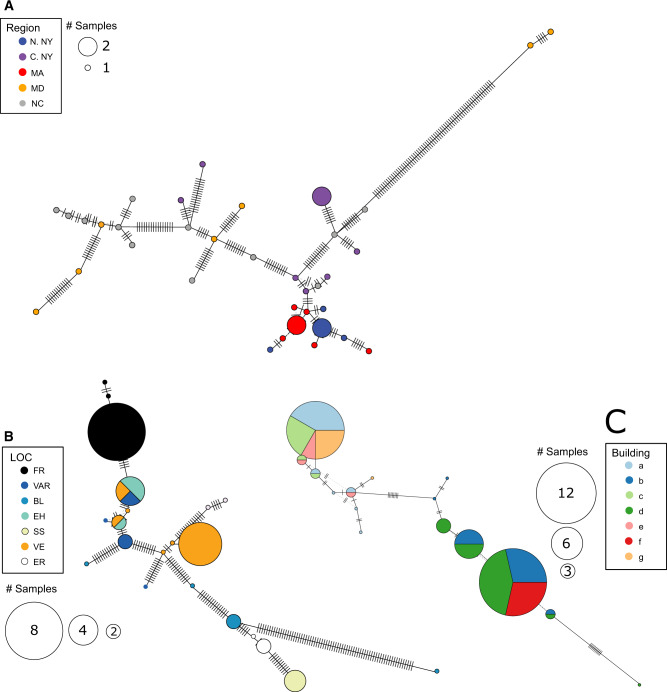
—Haplotype network of concatenated mitochondrial sequence across the (*A*) Eastern United States, (*B*) within Central New York, and (*C*) at a single site in Arnot Forest. Circle sizes correspond to the number of individuals with that haplotype. Colors indicate the sampling locations. Lines connect haplotypes to their most closely related neighbor. Bars represent mutational differences between neighboring haplotypes.

To further investigate patterns of genetic divergence, we constructed mitochondrial haplotype networks for the Eastern United States, Central New York, and within Arnot Forest (fig. 4). The distribution of haplotypes supports the findings from the MDS analysis. At the continental scale, haplotypes were not shared among Eastern US populations. Within Central New York, haplotypes were only shared among neighboring populations. At the local scale, mitochondrial differentiation was evident among buildings within a single clearing in the Arnot Forest.

### Dispersal

To look for evidence of differences in male and female dispersal distances, we compared nuclear and mitochondrial genetic diversity statistics. We calculated the difference between the observed and expected values for each pairwise comparison across the Eastern United States. The average expected value of *F*_ST(mito)_ was estimated as 0.24. This value was greater than the average observed value of 0.172 (one sample *t*-test, *t* = 2.49, df = 9, *P* = 0.03) indicating likely male-biased dispersal in these populations.

We estimated the mean parent–offspring axial dispersal distance using the slope of the regression line for the Eastern US comparison of *F*_ST_/(1 − *F*_ST_) against pairwise distance. This yields an estimated dispersal distance of *σ* = 761 m using the measurements of nest density from Arnot Forest and *σ* = 501 m using nest density estimates from Tsuchida et al. (2014). We repeated this measurement using the slope of the regression line from the Central New York comparison, although this regression line had a poor goodness of fit for our data. Using only the Central New York data, we estimate *σ* = 188 m based on the nest density from Arnot Forest and *σ* = 124 m using the nest density from Tsuchida et al. (2014).

### Comparison of *P. fuscatus* Genetic Variation with Additional *Polistes* Species

To further investigate the second genetic population identified in the fastSTRUCTURE analysis, we performed a second MDS analysis using all 204 samples collected across the Eastern United States (supplementary fig. S3, Supplementary Material online). Eigenvalues are given in supplementary table S3, Supplementary Material online. The MDS axis (C1) separated 24 samples from the remaining *P. fuscatus* individuals. Upper level MDS axes (2–10) were driven by variation within Central New York samples, likely caused by the overrepresentation of individuals from this region relative to the other regions in the analysis. These 24 samples were geographically widespread, and largely, but not entirely, corresponded with samples assigned >10% membership probability in group 2 or group 3 in the fastSTRUCTURE analysis (supplementary fig. S1, Supplementary Material online).

**Fig. 5.**
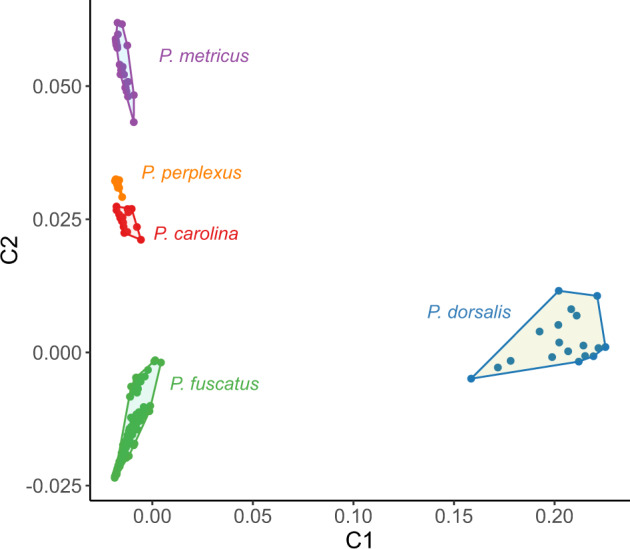
—Plot of the first two axes from a MDS analysis of whole-genome sequences for all *P. fuscatus* samples in this study and whole-genome sequences from four sympatric species of paper wasp.

A potential explanation for additional structure unrelated to geography is the inclusion of misidentified species within our samples. The northern range limit of *P. dorsalis* extends into Central New York and smaller *P. fuscatus* are commonly misclassified as *P. dorsalis* (Buck et al. 2008). Similarly, *P. metricus* occurs in Maryland and North Carolina and darker bodied *P. fuscatus* are less commonly misclassified as *P. metricus* (Buck et al. 2008). To account for this possibility, we combined the 204 Eastern US *P. fuscatus* from this study with 93 previously generated whole-genome sequences from four sympatric closely related *Polistes* species: *P. metricus*, *P. carolina*, *P. perplexus*, and *P. dorsalis* (Miller et al. 2020). An MDS analysis including these additional species clearly shows that these 24 individuals were not misclassified *P. dorsalis* or *P. metricus* (fig. 5). Interestingly, these 24 individuals show a slight separation along the second MDS axis (C2) from the other *P. fuscatus* samples. These individuals are also not recent hybrids between *P. fuscatus* and other species because they do not have an intermediate value in multidimensional space. There is no clear biological difference associated with these specimens and unraveling the cause of this genetic variance will require future study.

To test the contribution of these 24 samples to our findings, we repeated our calculations of IBD across the Eastern United States and within Central New York without these samples. Due to the smaller sample sizes of some populations, comparisons between Maryland and North Carolina were dropped from the Eastern US analysis, and all comparisons with Erin New York were dropped from the Central New York analysis. We find significant IBD in the nuclear genome across Eastern United States (*y* = 9.54 × 10^−5^*x* – 9.58 × 10^−3^, *R*^2^ = 0.68, *P* < 0.003) and a similar poor correlation between linearized genetic and geographic distance in Central New York (*y* = −0.001*x* + 0.08, *R*^2^ = 0.05, *P* = 0.32) (supplementary fig. S4, Supplementary Material online). There was no significant relationship between genetic distance and geographic distance using mitochondrial markers in the Eastern United States (*y* = 0.01*x* + 3.67, *R*^2^ = −0.08, *P* = 0.54) or in Central New York (*y* = −0.01*x* + 1.38, *R*^2^ = −0.26, *P* = 0.91). Calculating mean parent–offspring axial dispersal using the slope of regression line for the Eastern US comparison yields an estimate of *σ* = 578–879 m.

## Conclusion

Several lines of evidence support the presence of fine-scale genetic structure, and therefore limited dispersal, in Eastern US *P. fuscatus* populations. All studied populations had inbreeding coefficients >0. From the MDS clustering analysis, spatial genetic structure was evident across the Eastern United States, within Central New York, and at a site-specific scale in Arnot Forest, albeit with some overlap among populations. Notably, at the site-specific scale, individuals from Arnot Forest showed fine-scale differentiation across multiple years, indicating year-to-year fidelity of natal nest locations within a few meters at this site. The distribution of mitochondrial haplotypes additionally supports population structure across multiple scales. We detected distinct mitochondrial haplotypes in each regional subpopulation sampled across the Eastern United States. At the regional scale, haplotypes were shared only between neighboring sites, and at the site-specific scale, haplotype divergence was evident among buildings in some cases. Dispersal limitation is further supported by significant patterns of IBD detected in the nuclear and mitochondrial genomes across the Eastern United States and a trend toward IBD within Central New York. This fine-tuned degree of natal philopatry is consistent with previous mark-recapture findings (Klahn 1979).

Given the strong site fidelity indicated by lineage clustering within a single site in Arnot Forest, it is notable that our continental-scale analysis revealed low overall genetic differentiation between *P. fuscatus* populations sampled across the Eastern United States. The mean pairwise *F*_ST_ across all subpopulation comparisons was 0.053, with little genetic differentiation even among populations separated by hundreds of kilometers. Analyses of population structure predicted *K* = 1 or *K* = 2 demes as the best-supported model. The two predicted demes did not correspond to delineation between geographic regions but were interspersed within subpopulations (fig. 2 and supplementary fig. S1, Supplementary Material online). Similarly, analyses of patterns of genetic variation using MDS for the entire data set of 204 individuals found limited genetic differentiation within *P. fuscatus* across geographically widespread samples (fig. 5 and supplementary fig. S3, Supplementary Material online). Outlier individuals were largely, but not entirely shared between the two analyses. For example, individual 393 from Massachusetts was assigned to group 2 in the fastSTRUCTURE analysis (fig. 2) but was not one of the 24 samples separated in the MDS analyses (fig. 5 and supplementary fig. S3, Supplementary Material online). Similarly, all seven samples from Erin, New York were separated by the MDS analysis but only four of these samples were assigned >10% membership in group 2 or group 3 of the fastSTRUCTURE analysis (supplementary fig. S1, Supplementary Material online). Removing these 24 individuals did not change our overall conclusions. The loss of samples from Erin, New York reduced the magnitude of pairwise *F*_ST_/(1 – *F*_ST_) values in Central New York (fig. 3 and supplementary fig. S4, Supplementary Material online) but in both analyses, we did not observe a significant pattern of IBD. These findings of structure within geographically dispersed individuals are consistent with incomplete lineage sorting, the remnants of historical population structure, and/or introgression from a separate lineage rather than true population structure. Our detection of these patterns may be a result of the large number of SNPs used in this analysis and the sensitivity of whole-genome resequence data for identifying different evolutionary histories across the genome. Combined, these findings suggest that *P. fuscatus* is nearly panmictic across the Eastern United States.

Prior studies of population structure in *Polistes* have produced conflicting results regarding the extent of IBD and inbreeding in these species. Our findings echo these results leading to the question: How can we reconcile the detection of fine-scale structure at a single site with the low level of genetic differentiation observed at a continental scale? One possible explanation could be the presence of multiple dispersal strategies for *P. fuscatus* individuals.

Alternate female dispersal strategies may help to explain the observed high nest-site fidelity and simultaneously low overall population structure. Based on the observed pattern of IBD across the Eastern United States, we estimated a mean parent–offspring dispersal distance of 761 m. However, we suspect that the site used to estimate nest density in this study had a higher population density than other sites in Central New York, therefore 761 m is likely an underestimate of the average *P. fuscatus* dispersal distance. To put this estimated distance in context, *P. fuscatus* gynes forage on average within 48.1 m of their nest, but flights >220 m have been reported (Dew and Michener 1978). Although, as our results suggest, some gynes found new nests within a few meters of their natal nest, other individuals may be dispersing on the scale of hundreds to thousands of meters, thus accounting for the lack of broad-scale genetic structure.

These findings may also lead to a reinterpretation of results from past mark-recapture studies (Klahn 1979; Cervo and Turillazzi 1985; Gamboa et al. 1986; Sheehan et al. 2017). For example, Klahn (1979) recovered only 8.8% of marked female wasps in the spring following initial fall marking. This low rate of recovery was assumed to be largely the consequence of overwintering mortality but may instead represent long-distance migration by some females.

The link between dispersal and population structure is further complicated by the presence of two discrete decision periods for female dispersal. Dispersal events may occur during the nesting phase or during the mating phase (West 1967). When choosing a nest site, foundresses could employ alternate dispersal tactics depending on their cooperative breeding strategy. For example, females that choose to join cooperative associations with related individuals may remain near their natal nest, whereas those that found solitary nests may disperse further away thereby avoiding competition with their relatives. Female nesting philopatry has been confirmed through mark-recapture studies, but little is known about long-distance dispersal or mating phase dispersal in females. Similarly, multiple dispersal strategies could occur during the mating phase with females choosing to mate either near the natal nest or dispersing longer distances to mate, thereby leading to both local population structure and high rates of gene flow across the landscape. Disentangling these possibilities will require tracking patterns of female dispersal across different life stages.

Another explanation for the seemingly contradictory population structure findings across multiple scales could be alternate dispersal strategies between males and females. Longer dispersal distances in males have been reported in other Hymenopteran species (e.g., Ross and Shoemaker 1997; Clarke et al. 2002; Doums et al. 2002; Rüppell et al. 2003; Sundström et al. 2003). Adding to these studies, we find a discordance between population structure in nuclear and mitochondrial sequence, supporting longer-distance dispersal in males than females. Although mating behavior in paper wasps has received only limited attention, studies have shown that *P. fuscatus* males employ two different mating tactics. Males can join a lek and mate with females when they approach the lekking site, or males can patrol near foraging areas and attempt to mate opportunistically (Post and Jeanne 1983). Courtship is simple and mating success is driven by female choice (Miller, Legan, et al. 2018; Walton et al. 2020). Future studies of mating behavior and direct measurement of long-distance dispersal for males and females will help to clarify the complex relationship between *Polistes* life-history and dispersal. Outstanding questions relevant to dispersal include the following: 1) how far do males and females travel from their natal nest before mating? 2) Do males choose a single mating tactic or change courtship strategy over the season? 3) Do males stay at a single location or move among sites?

A third explanation for our results is that the low overall genetic divergence in *F*_ST_ between populations may be the result of mechanisms unrelated to contemporary dispersal. Within the last 10,000 years, *P. fuscatus* has undergone a northern range expansion into New York and Massachusetts due to melting of glaciers in these regions following the end of the last ice age. Prior to this time, *P. fuscatus* populations may have been smaller and more panmictic. Genetic divergence of contemporary populations could result from incomplete lineage sorting of ancestral alleles, or plausibly historical population structure following secondary contact of populations from different glacial refugia, although the use of multiple refugia has not been tested in this species. Additionally, the value of *F*_ST_ depends on both the variation within subpopulations and the total variation (Weir and Cockerham 1984). Mechanisms that decrease total variation, such as background selection, or mechanisms that increase variation within a subpopulation, such as elevated mutation rate or introgression, can reduce *F*_ST_ (Hartl and Clark 1997). However, these mechanisms must affect *P. fuscatus* over the entire range of the species to generate the observed pattern of low genetic divergence. Further investigation of genomic processes in paper wasps will help address the potential role of alternative mechanisms in generating low genetic divergence coupled with fine-scale population structure.

Findings from this study have important implications for theoretical models of the evolution of cooperation. Theories of social evolution predict that cooperation should tend to favor limited dispersal (Platt and Bever 2009) and promote linkage disequilibrium between cooperative traits and traits that enhance population structure (Powers et al. 2011). Hamilton (1964) first proposed that limited dispersal should promote cooperation by increasing relatedness between potential beneficiaries within kin neighborhoods. Subsequent models called to question Hamilton’s findings by introducing the counteracting effects of increased competition between neighboring kin (Murray and Gerrard 1984; Wilson et al. 1992). More recent theoretical models have recognized that even when accounting for these kin competition costs, in all but a few closed systems (e.g., fig wasps), cooperation and dispersal should coevolve in a positive feedback: Cooperative social systems favor limited dispersal, and the population viscosity resulting from limited dispersal makes cooperation more advantageous (Mitteldorf and Wilson 2000; Le Galliard et al. 2005; Hochberg et al. 2008; Platt and Bever 2009). A major empirical challenge for studies of social evolution has been to test these models of the predicted relationship between social systems and population structure. In addition to costs incurred through kin competition, social insects are especially vulnerable to the costs of limited dispersal wrought by inbreeding depression, due to the restriction of reproduction to specialized breeder castes (Chapman and Bourke 2001). In haplodiploid Hymenoptera, inbreeding is further detrimental because it leads to the production of infertile diploid males (Heimpel and de Boer 2008). However, if cooperatively breeding species employ multiple dispersal strategies, this may mitigate the negative effects of kin competition and inbreeding. Few theoretical papers have addressed the potential role of multiple dispersal strategies in the evolution of cooperative behavior (Mullon et al. 2018), including variation in behavioral strategies in future models may lead to new insights into the evolution and maintenance of cooperative breeding.

Our findings are consistent with the predictions made by the hypothesis that cooperative breeding is linked to fine-scale population structure. Specifically, we detect highly structured philopatry at the scale of a single site. However, this study has not explicitly tested this hypothesis as we lack information about cooperation rates in all sampled locations. Future comparative analyses are necessary to further probe evident fine-scale structure to more fully understand how cooperative nesting behavior relates to spatial genetic structure. If cooperative nesters have a greater tendency toward philopatry, we expect to find a pattern of greater fine-scale genetic structure between cooperatively nesting versus solitary nesting individuals. Similarly, if cooperatively breeding systems lead to a higher degree of natal philopatry, we expect to find correlations between foundress rates and population structure in future comparative studies between species of *Polistes* wasps that vary in their degree of cooperative nesting.

## Supplementary Material

evaa070_Supplementary_Data

## Notes

**Data deposition:** Sequence data are available at the NCBI sequence read archive in project PRJNA506242 and PRJNA482994.
