# Supplementary material for: Fine-Scale Population Structure but Limited Genetic Differentiation in a Cooperatively Breeding Paper Wasp
Source: Genome Biol Evol. 2020 Apr 9;12(5):701–14. doi: 10.1093/gbe/evaa070 (PMC7259676; doi:10.1093/gbe/evaa070)
Supplement: evaa070_Supplementary_Data [file evaa070_supplementary_data.zip › GBE Resubmission Supp Info_0325.docx]

Supporting Information

Figure S1: Analysis of population structure in *P. fuscatus* for demes K=3-6 calculated with the program fastSTRUCTURE. Each individual is represented as a single column and the name of the individual is listed at the bottom. Color within a column indicates the inferred posterior probability that the individual is a member of a particular cluster. Regions represented are Northern New York (N. NY), central New York (C. NY), Massachusetts (MA), Maryland (MD), and North Carolina (NC).

Figure S2: Analysis of population structure in *P. fuscatus* for demes K=2-6 calculated with the program STRUCTURE. Each individual is represented as a single column. Colors within a column indicate the inferred posterior probability that the individual is a member of a particular cluster. Individuals were downsampled so that subpopulations had an even distribution of samples. The names of each individual are given at the bottom of the figure. Regions represented are Northern New York (N. NY), central New York (C. NY), Massachusetts (MA), Maryland (MD), and North Carolina (NC).

Figure S3: Plot of the first two axes from a multidimensional scaling (MDS) analysis of whole genome sequences for all 204 individuals collected across the Eastern United States. The first axis of variation (C1) separates 24 geographically widespread individuals from the remainder of samples. Regions represented are Northern New York (N. NY), central New York (C. NY), Massachusetts (MA), Maryland (MD), and North Carolina (NC).

Figure S4: The comparison of linearized pairwise whole-genome divergence versus geographic distance was repeated after removing the 24 individuals with values C1 < -0.02 from Figure S3. After removing these individuals, we still observed a significant pattern of isolation by distance (IBD) across the (A) Eastern USA using nuclear markers (y = 9.54 * 10^-5^x – 9.58 * 10^-3^, R^2^ = 0.68, p < 0.003) but (B) no significant correlation within the Central New York region (C. NY) (y = -0.001x + 0.08 , R^2^ = 0.05, p = 0.32). There was no significant correlation between pairwise mitochondrial divergence versus geographic distance across the (C) Eastern USA (y = 0.01x + 3.67; R^2^ =-0.08, p = 0.54) and (D) within the Central New York region (y = -0.01x + 1.38; R^2^ = -0.26, p = 0.91).

Table S1: Previous studies on population structure in *Polistes* wasps. Mean foundress number (f*_avg_*) as reported in Miller *et al.* 2018a, Location (Loc.), radius of total study population in kilometers(*r*P), average radius of defined subpopulations in kilometers (*r*SP), month(s) sampled, types and numbers of genetic loci used (P = protein-coding, RS = ribosomal DNA restriction site, M = microsatellite, COI = mitochondrial gene, WGS = whole genome sequence), number of subpopulations (Ns), number of nests (Nn), number of individuals (Ni), mitochondrial nucleotide diversity (π*mt*), inbreeding coefficient (*F*IS), fixation index (*F*ST), mitochondrial fixation index (Φ*S T* ), and detection of IBD (NS = no significant IBD, S = significant IBD). Data not reported (NR).

Table S2: Sample information for the individuals sequenced in this study. Date indicates the date the sample was collected. Regions are central New York (C. NY), Northern New York (N. NY), Massachusetts (MA), Maryland (MD), and North Carolina (NC). Samples were collected either on a nest (nest) or while foraging (wing). SRA ID is the accession number for the sample at the NCBI sequence read archive. Depth is the average sequencing depth.

See Attached.

Table S3: Eigenvalues and the percentage variance explained by each eigenvalue (%) for the Eastern USA (subset), Central NY, and Arnot Forest MDS plots in Figure 1; the Eastern USA (all) MDS plot in Figure S3; and the Across Species MDS plot in Figure 5.

Table S4: Measures of pairwise F_ST_ for the genome and for mitochondrial markers between populations across the Eastern USA and populations within Central New York. Abbreviations for populations are given in Table S2.
